# Factors promoting or limiting self-determined childbirth: midwives’ perspectives in Germany: Marie Tallarek

**DOI:** 10.1093/eurpub/ckac131.423

**Published:** 2022-10-25

**Authors:** M Tallarek, J Spallek

**Affiliations:** Department of Public Health, Brandenburg University of Technology Cottbus-Senftenberg, Senftenberg, Germany

## Abstract

**Background:**

Claims for self-determined childbirth (SDC) have gained increasing scientific, political and societal attention. However, research on SDC is limited. This study analyzes and compares midwives’ experiences and perspectives on factors that promote or limit SDC in hospitals, birthing centers and during home births in Germany. We argue that these insights are essential in order to foster self-determination and to avoid its violation.

**Methods:**

A qualitative case study was conducted based on semi-structured face-to-face interviews with midwives working in hospitals, birthing centers, and offering home births in Germany. In total, nine interviews were conducted in 2021 and have been audiotaped, transcribed, anonymized and analyzed by use of Thematic Analysis.

**Results:**

The results indicate eight inter-related categories, each of which imply promoting and limiting factors: 1) Structural/ legal conditions; 2) Perception of birth (e. g. as natural or medical process; required competence and control); 3) Trust and atmosphere; 4) Getting acquainted/relationship building; 5) Birthing person’s socioeconomic position; 6) Birthing person’s preparation/ education; 7) Birthing person’s capability of decision making and expression; and 8) Behavior of accompanying persons. Moreover, we identified midwives’ strategies to extend possibilities of choice. Several factors clearly differ depending on the birth setting.

**Conclusions:**

The opportunities for SDC seem to differ according to the setting (e.g. institutional routines), inter-personal relations (e.g. getting acquainted, trust), and individual factors (e.g. socioeconomic position, capabilities). Hence, political, institutional and individual strategies may support SDC in consideration of the above factors. Measures may, among others, include the improvement of information processes, the reduction of economic barriers, relationship building before and during birth as well as respective structural adjustments.

**Key messages:**

• Self-determination (SD) in childbirth is influenced by several factors at individual, inter-personal, institutional and macro level.

• Individual, institutional and political strategies may support SD.

